# Creatine formulations and repeated sprint training: effects on physical and physiological adaptations in soccer players during the short-term preparation phase

**DOI:** 10.1080/15502783.2026.2721147

**Published:** 2026-09-10

**Authors:** Changyuan Duan, Zaitao Wang, Qianjin Wang

**Affiliations:** a Sports Science Department, Wenzhou Medical University, Wenzhou, Zhejiang, People's Republic of China

**Keywords:** Interval intervention, supplementation, physical performance, football

## Abstract

**Background:**

This research examined the impact of various creatine (Cr) supplementation formulations during a 4-week preparatory phase on the efficacy of repeated sprint training (RST) and on improvements in physical and physiological performance in male soccer athletes.

**Methods:**

A total of forty collegiate young soccer players volunteered for the study. They were randomly divided into four groups: creatine monohydrate (Cr-Mon, *n* = 10), creatine ethyl ester (Cr-Ee, *n* = 10), creatine hydrochloride (Cr-Hcl, *n* = 10), and placebo (PL, *n* = 10). All athletes engaged in a 4-week training intervention, three times a week (i.e. 12 sessions), and physical (countermovement vertical jump [CMVJ], 20-m sprint, and L-run) and physiological (Wingate anaerobic power and incremental exercise tests) performance were assessed both before and after the training period.

**Results:**

All training groups improved physical and physiological performance following the 4-week intervention period (*p* < 0.05). Additionally, the groups receiving the Cr supplement exhibited significantly greater changes (*p* = 0.001) in physical performance, peak power output, and fatigue index compared to the PL group. However, no significant differences were found among the groups regarding mean power output and VO2max. Notably, the Ee and Hcl forms of Cr showed superior gains (*p* < 0.05) in physical performance and peak power compared to the Mon form after the 4-week intervention.

**Conclusions:**

The results underscore the importance of Cr ingestion in enhancing adaptations for short-term physical performance tasks, highlighting the effectiveness of the Ee and Hcl forms in further improving the CMVJ, 20-m sprint, L-run, and peak power among soccer players during the short-term preparation phase.

## 1. Introduction

Soccer is a high-intensity, intermittent team sport that alternates short bursts of intense activity with lower-intensity movements [1]. Players engage in various explosive actions and directional changes that require both muscular power and speed, along with aerobic and anaerobic fitness, which are essential for success in matches [2,3]. Repeated sprint training (RST) has proven to be an effective training strategy for improving muscular power, jumping ability, sprinting speed, and agility among soccer players [4–7]. This training method not only leads to more effective aerobic and anaerobic performance adaptations but also enhances enjoyment by reducing the perceived intensity of effort for the players [8].

Alongside the importance of an effective training programme for soccer players in the preparation phase of the annual training plan [9], many strength and conditioning coaches incorporate sports supplements to promote additional gains in physical performance during the short-term preparation phase [10]. Creatine (Cr) is recognised as one of the most prevalent nutritional ergogenic aids for improving the physical performance of soccer players [11,12]. Numerous studies have indicated that Cr supplementation enhances intramuscular creatine concentrations [13], potentially explaining improvements in high-intensity exercise performance [14] and facilitating greater training adaptations [15].

Many forms of creatine have been developed, including monohydrate, magnesium chelate, citrate, nitrate, ethyl ester, and hydrochloride, mainly to improve muscle creatine storage and athletic performance [13]. Among these, creatine monohydrate (Cr-Mon) is widely regarded as the standard form of supplementation because strong evidence supports its effectiveness in increasing muscle creatine and phosphocreatine (PCr) levels, which help speed ATP resynthesis during high-intensity exercise [13–15]. Despite the well-established effectiveness of Cr-Mon, some manufacturers have attempted to improve its solubility and claimed bioavailability through chemical modifications such as esterification or acid salt formation [16–18]. For example, creatine ethyl ester (Cr-Ee) was developed to reduce hydrophilicity and improve passage across the muscle cell membrane [17,19]. Similarly, creatine hydrochloride (Cr-Hcl) has been promoted as having greater water solubility than Cr-Mon [18,20,21]. However, these theoretical advantages in solubility and permeability do not necessarily lead to better clinical outcomes. Current evidence suggests that the bioavailability of Cr-Mon is already very high, and direct evidence showing that these newer forms are superior to Cr-Mon remains limited and inconsistent [22]. Therefore, although alternative forms differ in their physicochemical properties, their practical value for improving athletic performance is still under debate.

While previous studies have documented the ergogenic benefits of various forms of Cr supplementation in enhancing athletes' physical performance [11–20], there is a lack of published evidence demonstrating the superiority of each Cr type in relation to performance adaptations in soccer players. Research comparing Cr-Mon to Cr-Ee and Cr-Mon to Cr-Hcl during resistance training has yielded inconsistent findings [16–20]. Furthermore, the acute effects of these Cr types and the focus on a single training modality (i.e. resistance training) have not contributed to a clearer understanding of the advantages of each Cr form over the others [11,12]. It is essential to address these gaps to gain a thorough insight into the impact of different Cr supplementation types on soccer players' performance. Consequently, this study aims to evaluate the effects of short-term (i.e. 28 days) Cr supplementation with various formulations, combined with RST, on physical and physiological performance adaptations in young male soccer players with aiming to clarify the relative effectiveness of each Cr type, including Cr-Mon, which emphasises the combination of Cr and water molecules; Cr-Ee, which aims to enhance bioavailability through esterification; and Cr-Hcl, which promotes absorption by forming a salt. We hypothesised that using more bioavailable and absorbable Cr could enhance RST effectiveness, resulting in greater physical performance improvements for soccer players.

## 2. Materials and methods

### 2.1. Ethical statements

Each participant received comprehensive information regarding the potential risks and benefits associated with the study. The experimental procedures employed in this research were approved by the University's Ethics Committee (2023.04.26784) and were conducted in accordance with the latest version of the Declaration of Helsinki.

### 2.2. Sample size calculation and randomisation

An *a priori* power analysis was performed using G*Power (version 3.1.9, Universität Düsseldorf, Germany) for a repeated measures ANOVA (within-between interaction). While previous studies have occasionally identified small effect sizes (e.g. 0.21) in general populations [19,20], this study was designed to detect moderate-to-large effects (e.g. 0.35), which are considered more representative of physiologically meaningful performance adaptations in collegiate soccer players. Based on an alpha of 0.05 and a power of 0.95, a sample size of 40 was determined to be robust for detecting such relevant outcomes. This sample size is consistent with current literature in soccer science [5,11], which frequently balances statistical rigour with the inherent logistical challenges of recruiting trained athletes. Participants were evenly divided into four groups of ten, reflecting the number of outfield players on a soccer team (excluding the goalkeeper). To ensure rigorous methodological standards, group allocation was performed via a computerised random number generator (simple randomization, 1:1:1:1 ratio), guaranteeing that assignments remained concealed and unpredictable to both the research team and the participants.

### 2.3. Participants

In this study, 40 collegiate male soccer players with similar fitness levels and training practices during the pre-season volunteered and were assigned to one of four groups: creatine monohydrate (Cr-Mon), creatine ethyl ester (Cr-Ee), creatine hydrochloride (Cr-Hcl), and a placebo (PL) (Table 1). To be eligible for inclusion, players had to adhere to specific criteria: 1) they should have experience with various forms of interval training but had not participated in RST for three months before the study's start, 2) they should be free from any medical or orthopaedic conditions that could impact their participation or performance, as confirmed by a sports medicine physician [23], 3) they were required to be classified as trained athletes (i.e. Tier 2) [24], and) they should not have taken any Cr or other sport supplements for at least six months before their involvement in the study.

**Table 1.** Participants characteristics' (mean ± SD).

Characteristics	Cr-Mon	Cr-Ee	Cr-Hcl	PL
Age (y)	20.5 ± 1.5	20.6 ± 1.6	20.7 ± 1.5	20.4 ± 1.7
Height (cm)	174.4 ± 3.9	173.7 ± 4.8	175.2 ± 3.3	174.8 ± 4.1
Body mass (kg)	74.2 ± 3.3	73.7 ± 3.6	73.3 ± 3.9	74.7 ± 3.1
Soccer training age (y)	5.8 ± 0.8	5.7 ± 0.6	6.1 ± 1.1	5.5 ± 0.9

Each groups consisted of 10 athletes including 4 defenders, 4 midfielders, and 2 forwarder.

### 2.4. Experimental design

A pre/post-test design was used during the preparation phase of this study. The study lasted six weeks and included one week for familiarisation with the study's design and procedures, as well as anthropometric measurements (height measured with a wall-mounted stadiometer [Seca 222, ± 0.5 cm] and body mass assessed with a digital scale [Tanita, ± 0.1 kg]). This was followed by one week for the pre-test, four weeks for the RST intervention and Cr supplementation, and one week for the post-test. Physical and physiological performance measurements were taken over three days, with 48-hour intervals between testing sessions: on day one (i.e. Monday), countermovement vertical jump (CMVJ) and 20-m sprint were measured; on day two (i.e. Wednesday), L-run change of direction ability and the Wingate anaerobic power test were evaluated; and on day three (i.e. Friday), cardiorespiratory fitness was assessed through an incremental exercise test. To facilitate adequate recovery, 10-minute rest periods were incorporated between exercise performance tests. After the pre-test, all athletes engaged in a four-week soccer training programme (five days a week), with the RST included prior to regular soccer practices on Mondays, Wednesdays, and Fridays. Participants consumed their designated Cr type or a PL in a double-blind manner throughout the 28-day intervention period. Upon completion of the training, the sequence and order of the pre-intervention tests were repeated for the post-test. Given that all tactical and technical soccer training sessions were scheduled for the afternoon, it was determined that all testing and RST sessions would also take place during this time to minimise the influence of circadian rhythms on performance. The CMVJ, 20-m sprint, and L-run assessments were conducted on a soccer grass pitch. At the same time, the Wingate anaerobic power and incremental exercise tests were performed in a controlled laboratory setting with temperatures ranging from 27 to 29 °C and humidity levels between 45 and 55%, all under the supervision of the same examiner. Participants were instructed to wear coordinated footwear, standard training shorts, and T-shirts for both the pre-test and post-test evaluations.

### 2.5. Cr supplementation

During the intervention, participants in the Cr and PL groups consumed 5 g daily of either Cr-monohydrate, Cr-ethyl ester, Cr-hydrochloride, or polydextrose. This 5 g dosage was consistently maintained across all creatine groups throughout the study. This specific amount was selected because it aligns with established sports nutrition research, which identifies 5 g as an effective daily dose for maintaining muscle creatine saturation in individuals weighing approximately 75 kg [13,15,17–20]. While a daily maintenance dose of 5 g, without an initial loading phase of 20 g/day, may take approximately 3 to 4 weeks to achieve maximal intramuscular creatine saturation [22], this protocol was deliberately chosen to assess the cumulative ergogenic effects of these formulations under conditions that enhance gastrointestinal tolerability and reflect practical long-term supplementation use. In addition, the same 5 g dose was used across all experimental groups to allow a standardised comparison of commercially available products. However, we acknowledge that because creatine monohydrate, creatine ester, and creatine hydrochloride differ in chemical structure and molar mass, the exact amount of elemental creatine provided was not identical across formulations. Nevertheless, this approach was necessary to minimise confounding variables associated with differing powder volumes, thereby ensuring a standardised comparison of formulation efficacy under consistent, real-world training conditions. Both creatine and placebo powders were encapsulated to ensure identical appearance and taste. Participants were instructed to consume their assigned capsules with 200 mL of water 30 minutes before training. On non-training days, the dose was taken at the same time in the afternoon to maintain consistency. To ensure a double-blind design, capsules were identical in appearance and lacked any identifying labels; consequently, neither the researchers nor the participants were aware of the group assignments until the study’s conclusion. Compliance was monitored through the weekly distribution and collection of supplement packets, along with the completion of intake forms. Additionally, the researchers conducted weekly phone calls to remind participants of the protocol and verify adherence throughout the four weeks.

### 2.6. Training intervention

All athletes engaged in a five-day soccer training programme aimed at the preparation phase. Each session lasted between 110 and 120 minutes, comprising a 15-minute warm-up, followed by 85 to 95 minutes of primary training activities, including tactical and technical drills, small-sided games, and simulated competitive matches, and concluding with a 10-minute cool-down. Training loads were adjusted according to players' specific positions, and they maintained consistent training habits throughout the intervention period. Furthermore, the athletes participated in an RST programme involving 3 sets of 12 maximal-effort (i.e. *all-out*) 5-second sprints, with 20 seconds of active rest between trials (1:4 ratio). A 3-minute rest period was implemented between sets to facilitate recovery. This programme was executed three times a week, with 72-hour breaks between sessions. All RST sessions took place on a grass soccer pitch and were closely monitored by a strength and conditioning coach along with a researcher to ensure compliance with the training protocols [4–6,8].

### 2.7. Testing procedure

Before testing, participants received detailed instructions, including obtaining at least 8 hours of quality sleep and wearing the same type of footwear for both the pre- and post-tests, as verified through personal interviews. They were also advised to maintain their usual daily activities and dietary habits, refraining from using any performance-enhancing supplements. Each participant completed a 15-minute general warm-up routine consisting of 5 minutes of light jogging, followed by 5 minutes of static and dynamic stretching exercises, and concluding with 5 minutes of low-intensity sprints. Additionally, a specific warm-up was conducted, involving 3-5 trials at submaximal effort for each physical performance test.

**Figure 1.**
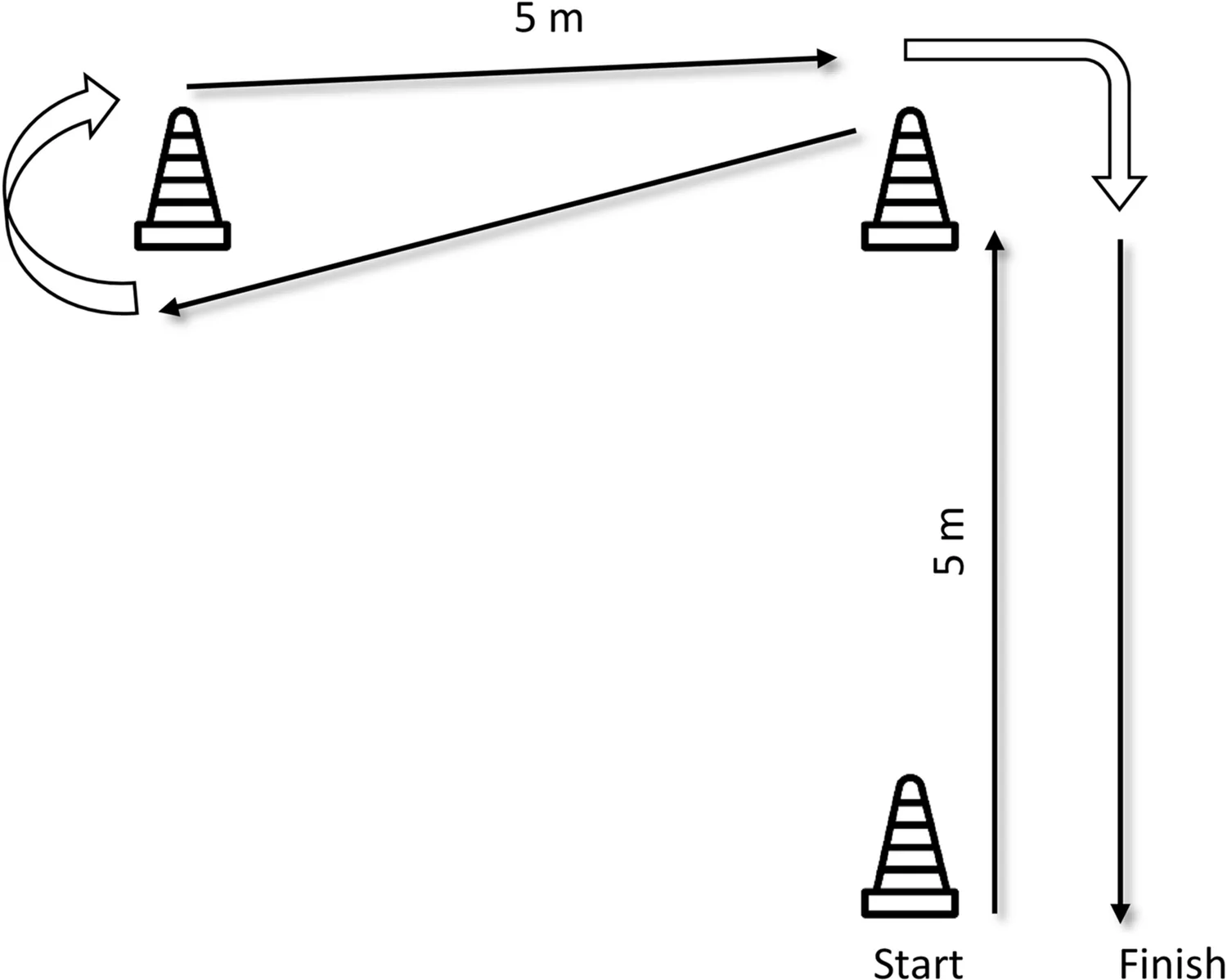
L-CODS procedure.

*Physical performance measurement procedures*: A wall-mounted vertical jump tester (Power System, USA) was used to assess the CMVJ. This test began with participants standing straight. When they were ready, they flexed their knees to lower themselves to a depth of their choice (up to 90º). They then extended their knees to jump as high as they could. At the highest point of their jump, participants made contact with the vanes on the Vertec. The difference between the highest vane touched during the jump and the participant's standing reach was recorded [4]. The duration of the 20-m linear sprint was recorded to the nearest 0.01 second utilising a single-beam timing gate system (Brower Timing System, USA). Participants began the sprint test from a stationary position, with the toe of their dominant foot placed forward and behind the starting line. Timing commenced when the participant voluntarily initiated the sprint. The timing gates were set up at the start (0.3 m in front of the starting line) and at the 20-m finish point, positioned approximately 0.7 m above the ground (at hip height) to accurately capture trunk movement and prevent false triggers from limb movements [6]. For the L-run, the timing system and procedures were consistent with those used for the 20-m sprint, except that participants were required to run straight while executing multiple rapid directional changes, as described in Figure 1 [25]. The most favourable score from the three trials was noted for subsequent analysis. The reliability coefficient (ICC) for repeated measurements at CMVJ, 20-m sprint, and L-run were 0.95, 0.92, and 0.96, respectively.

*Physiological performance measurement procedures*: A cycle ergometer (Ergomedics 874, Monark, Sweden) was utilised to perform the Wingate anaerobic power test. Following a 5-minute warm-up, participants were instructed to pedal as rapidly as possible for 30 seconds against a resistance determined by multiplying their body weight in kilograms by 0.075. Peak power was determined by averaging power output over a 5-second interval, which typically represents maximum performance [26] and is usually observed in the initial 5 seconds of the test. The mean power was calculated as the average power output over the entire 30-second period. During the test, participants received verbal encouragement to exert their utmost effort throughout the full 30 seconds [4,27]. In addition to peak and mean power outputs, the fatigue index (FI) was calculated to quantify the rate of power decline during the 30-s Wingate test. FI was determined using the formula: FI (%) = [(peak power − minimum power)/peak power] × 100. This index provides an estimate of the ability to maintain power output throughout the test and is commonly used as an indicator of anaerobic fatigue [27]. To evaluate maximum oxygen consumption (VO_2max_), a graded exercise test was performed on a treadmill (SportsArt Fitness, T645L, Everett, WA 98203, USA), employing a breath-by-breath gas collection system (Hans Rudolph Inc., USA). The testing protocol commenced at 8 km/h, with increments of 1 km/h every 3 minutes until participants reached voluntary exhaustion. Several criteria were utilised to confirm the achievement of VO_2max_, which included: a) a stable VO_2_ level despite increased workload, b) a respiratory exchange ratio exceeding 1.10, c) a blood lactate concentration of 8 mmol/L or more, d) a maximum heart rate of at least 95% of the age-predicted maximum (220 minus age), and e) the experience of voluntary exhaustion [28].

### 2.8. Control of diet

To reduce the potential effect of dietary status on performance outcomes, a structured dietary monitoring protocol was implemented. Before the intervention, participants were asked to complete a 3-day dietary record, including two weekdays and one weekend day. These records were analysed using Nutritionist Pro™ software (version IV) to estimate habitual energy intake and macronutrient consumption. Based on the results, participants were instructed to maintain their usual dietary habits throughout the study.

More specifically, they were asked to follow an isocaloric diet of approximately 3000 kcal per day, with a macronutrient distribution of 20% protein, 60% carbohydrates, and 20% fat. Participants were also instructed not to use any new nutritional or performance-enhancing supplements during the study period. To support compliance, the research team provided regular scheduled reminders to help participants maintain their prescribed dietary intake and usual eating habits. Any major dietary deviations or use of prohibited supplements were monitored through ongoing follow-up communication to keep nutritional variables as consistent as possible throughout the intervention.

### 2.9. Statistical analysis

The data are presented as mean ± standard deviation (SD). Normality was assessed using the Shapiro-Wilk test for both pre-test and post-test values, and homogeneity of variances was examined with Levene’s test. Differences among groups were analysed using a two-factor repeated-measures analysis of variance (ANOVA) [time (2) × group (4)], followed by Bonferroni post hoc tests when appropriate.

Effect sizes (ES) were calculated with 95% confidence intervals (CI) to assess the magnitude of the training effects. According to the classification proposed by Hopkins et al. [29], ES values were interpreted as follows: < 0.2, trivial; 0.2–0.6, small; 0.6–1.2, moderate; 1.2–2.0, large; 2.0–4.0, very large; and > 4.0, nearly perfect. Individual percentage changes were also calculated for each variable over time using the formula: ∆% = (post - pre)/pre × 100. In addition, one-way ANOVA was used to compare the magnitude of percentage changes (∆%) among groups, with Bonferroni post hoc tests applied for pairwise comparisons. Statistical significance was set at *p* < 0.05. All analyses were performed using SPSS version 21.0 (SPSS, Chicago, IL, USA). If you want, I can also make this more concise for a Methods section or more polished for a high-impact journal style.

## 3. Results

The players completed all training sessions, resulting in a success rate of 100%, as confirmed by the personal attendance records. Moreover, no injuries related to the testing and training protocols were reported among the participants, and no gastrointestinal problems were associated with Cr or PL supplementation.

**Figure 2.**
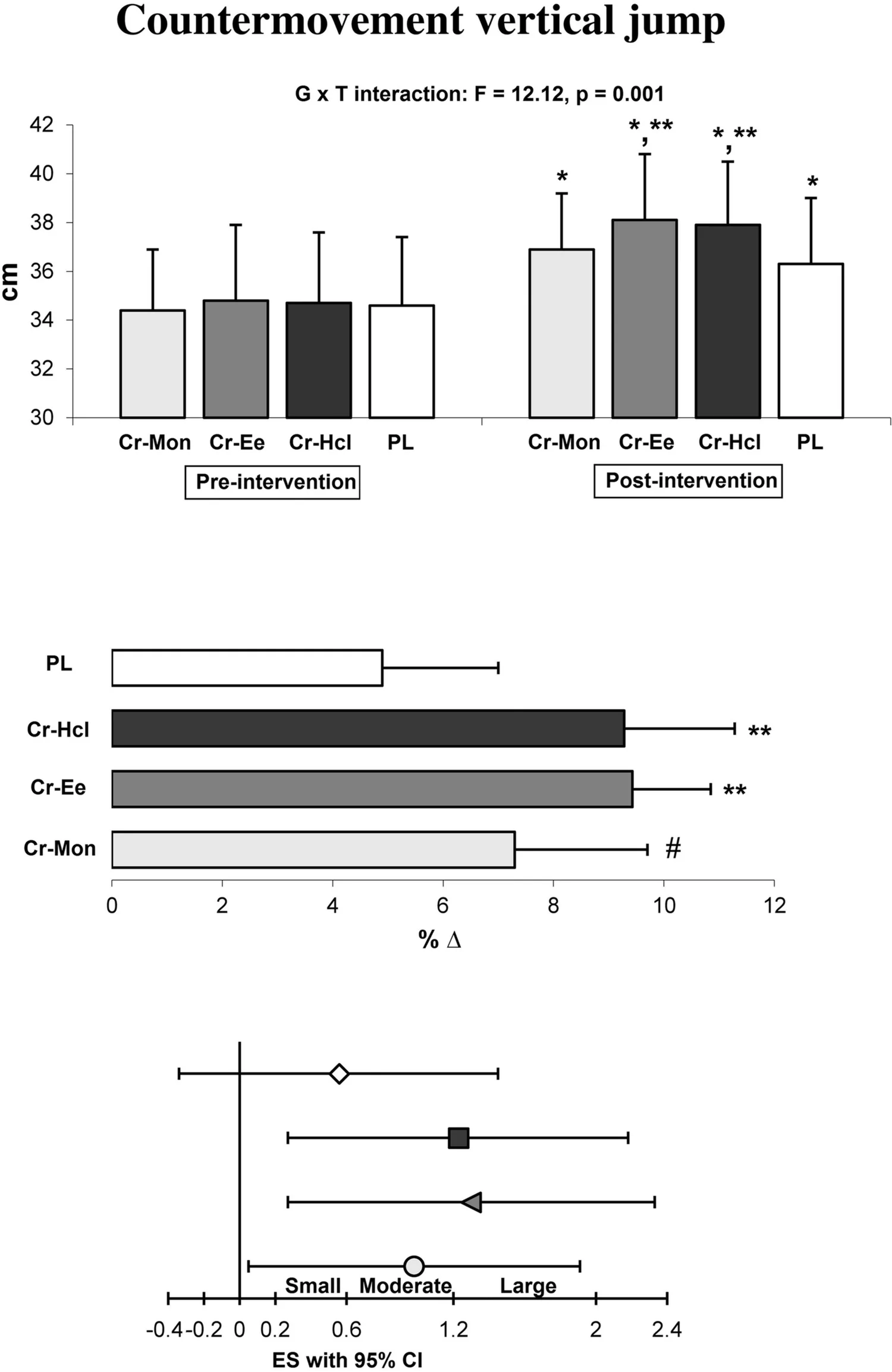
Changes in the countermovement jump through the 4-week intervention period. *denotes significant differences compared with the pre, **denotes significant differences compared with the Cr-Mon and PL, ^#^denotes significant differences compared with the PL.

**Figure 3.**
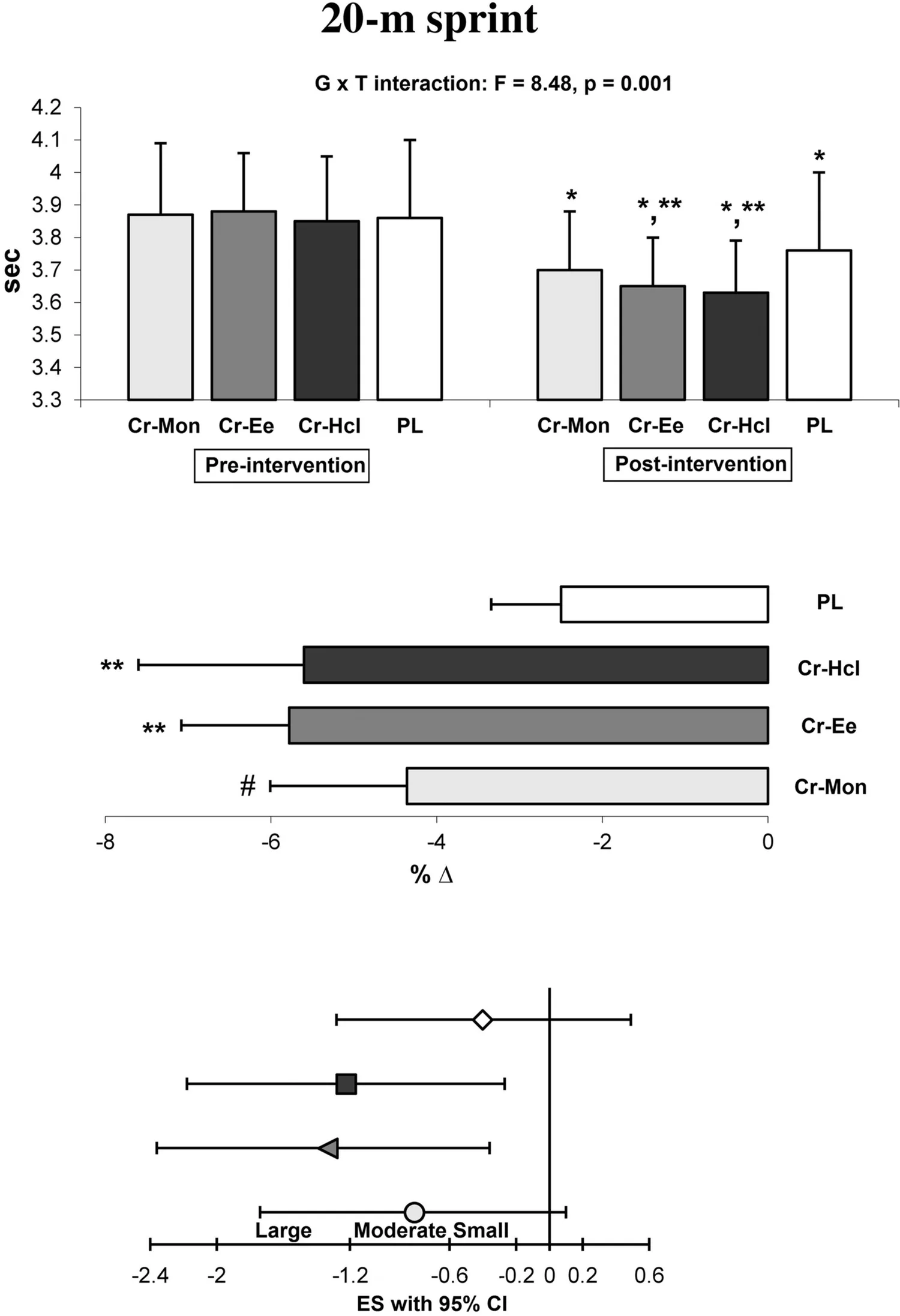
Changes in the 20-m sprint through the 4-week intervention period. *denotes significant differences compared with the pre, **denotes significant differences compared with the Cr-Mon and PL, ^#^denotes significant differences compared with the PL.

**Figure 4.**
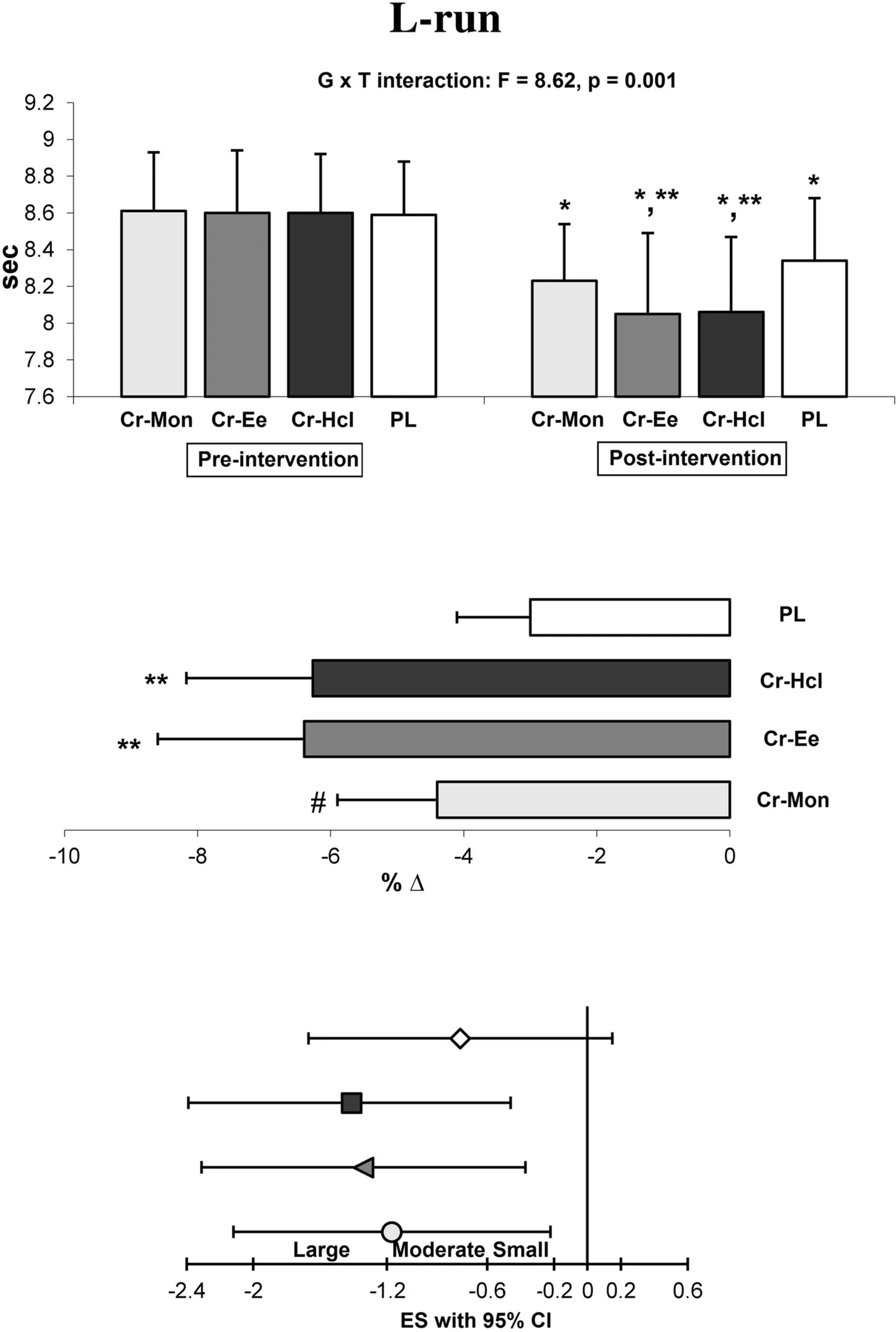
Changes in the L-run through the 4-week intervention period. *denotes significant differences compared with the pre, **denotes significant differences compared with the Cr-Mon and PL, ^#^denotes significant differences compared with the PL.

**Figure 5.**
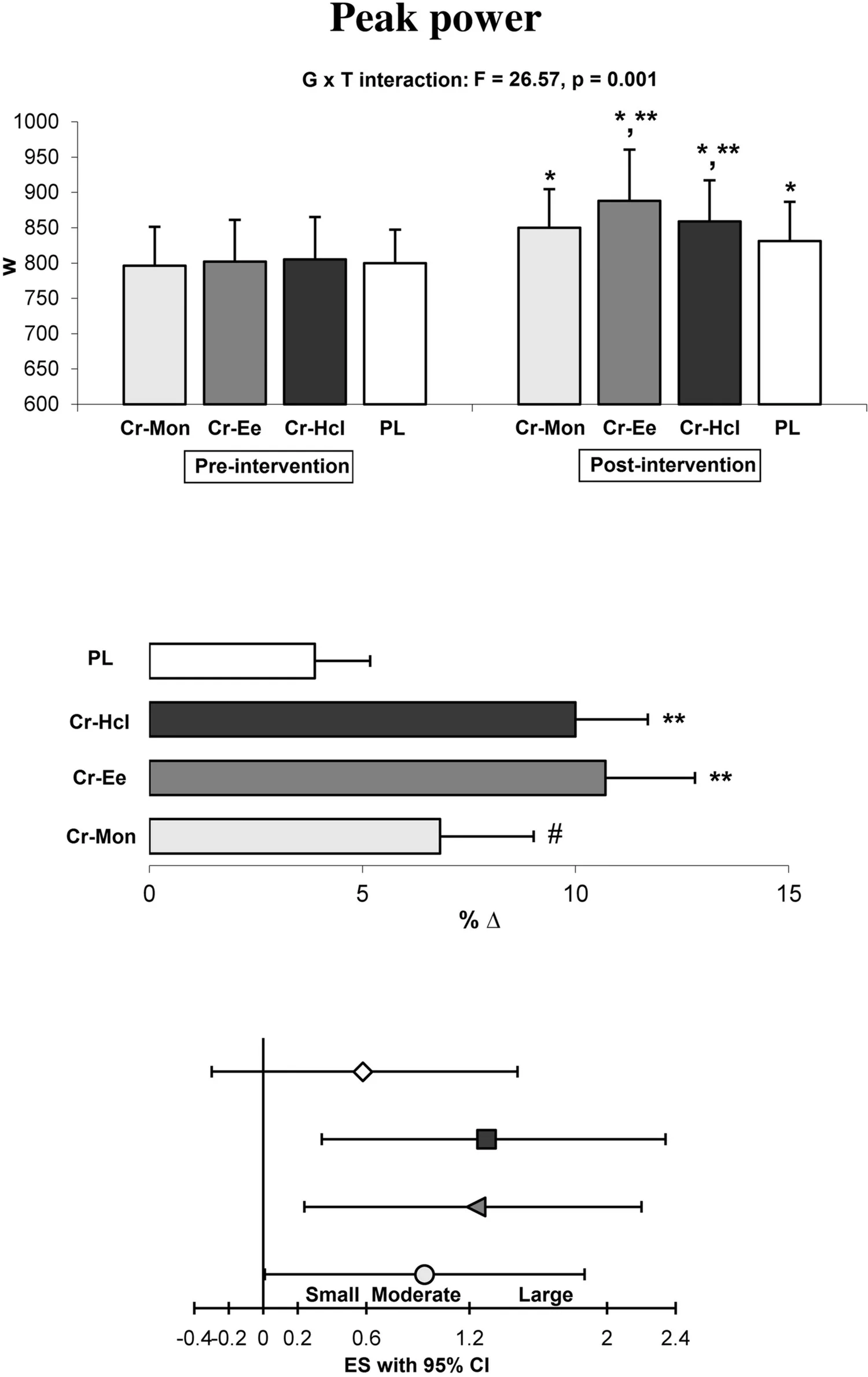
Changes in the peak power through the 4-week intervention period. *denotes significant differences compared with the pre, **denotes significant differences compared with the Cr-Mon and PL, ^#^denotes significant differences compared with the PL.

**Figure 6.**
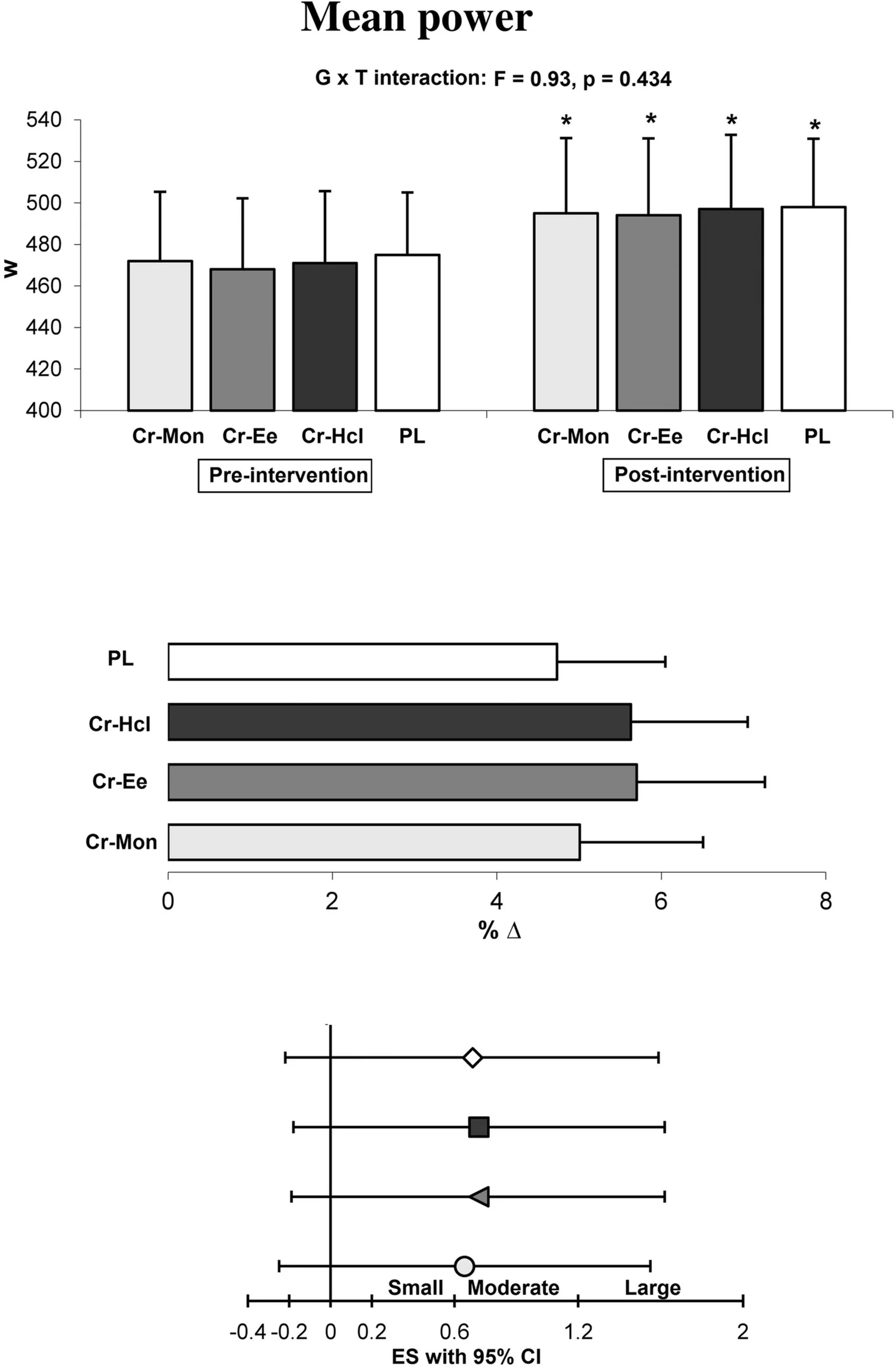
Changes in the mean power through the 4-week intervention period. *denotes significant differences compared with the pre.

**Figure 7.**
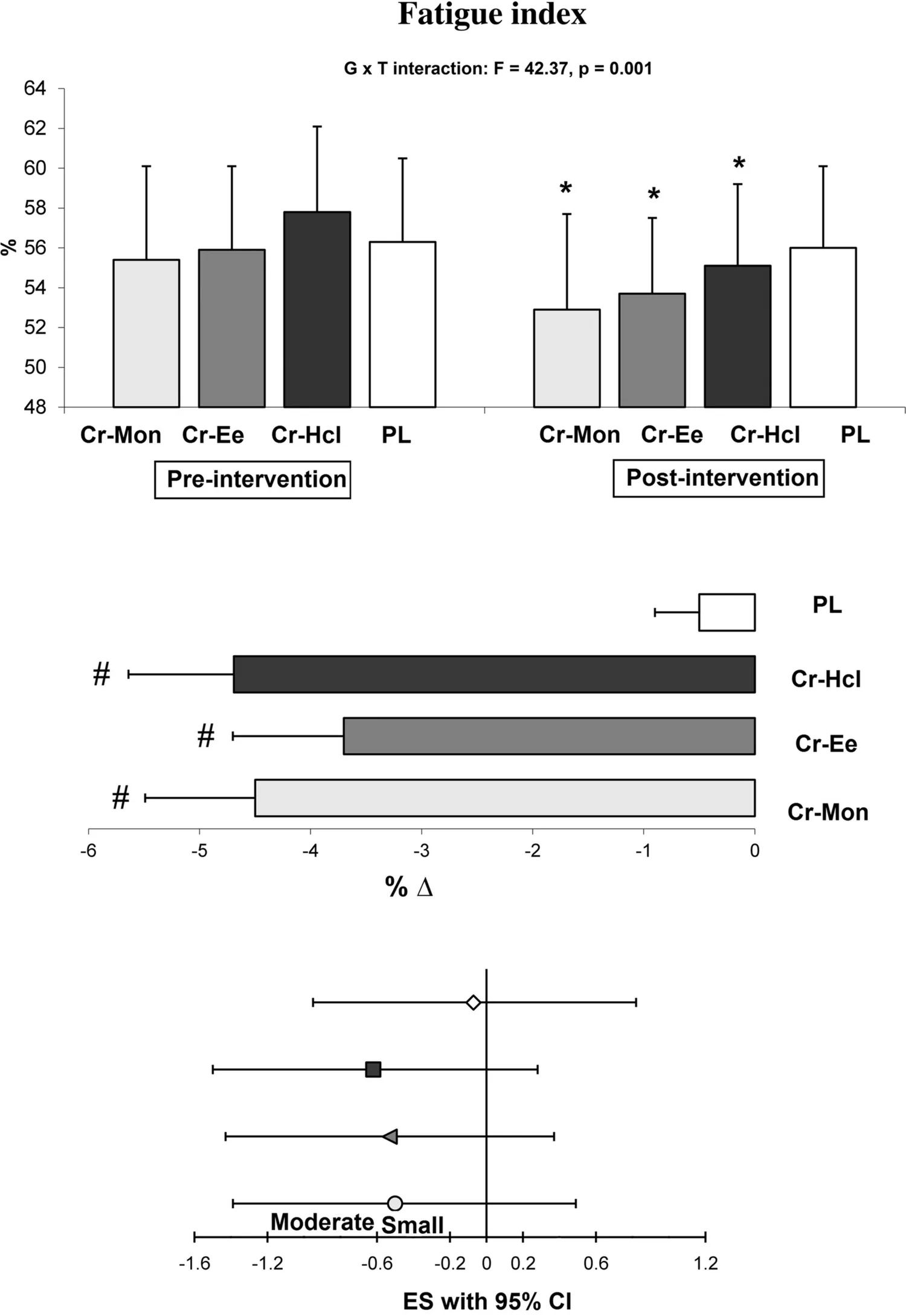
Changes in the fatigue index through the 4-week intervention period. *denotes significant differences compared with the pre, ^#^denotes significant differences compared with the PL.

**Figure 8.**
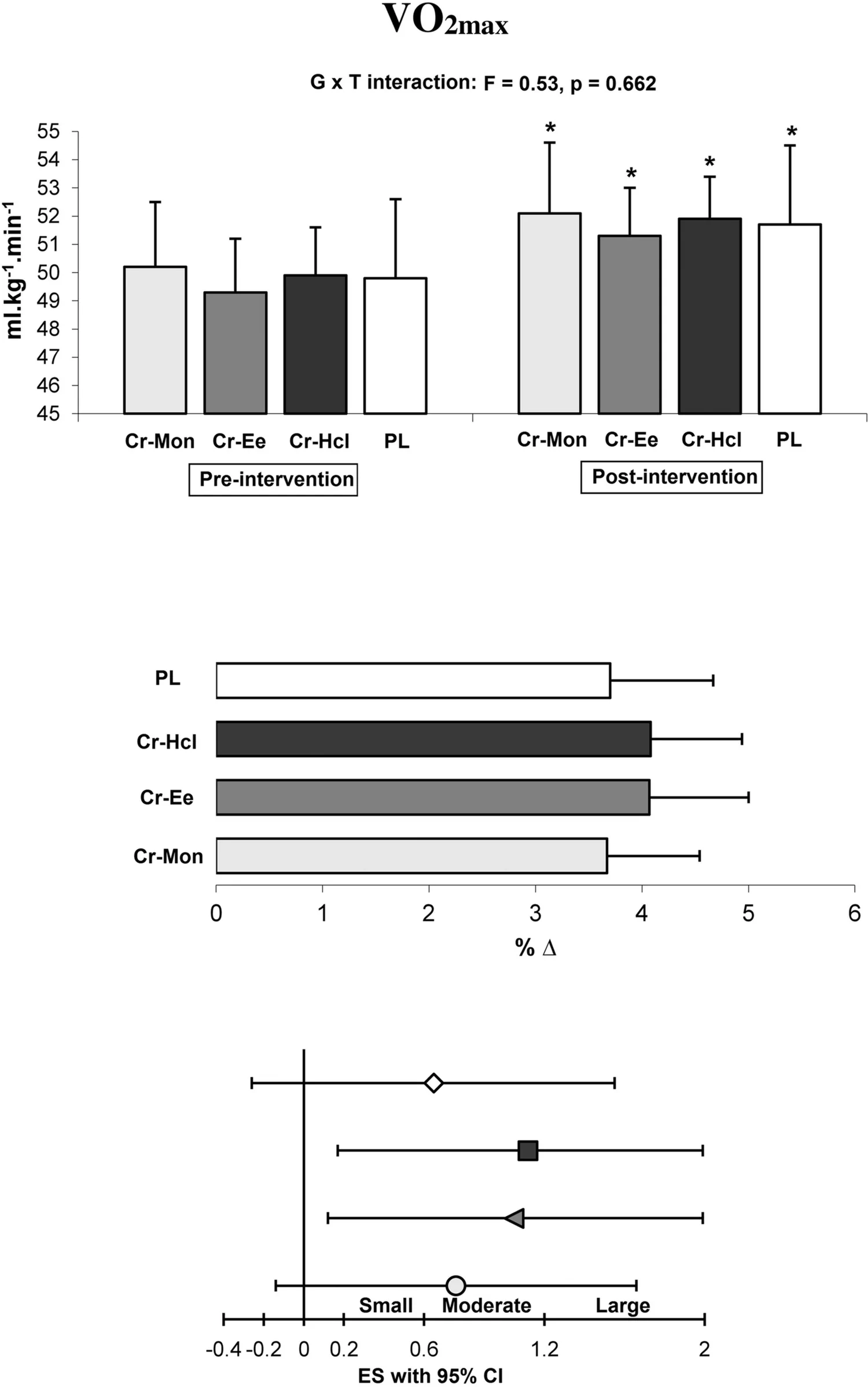
Changes in the VO_2max_ through the 4-week intervention period. *denotes significant differences compared with the pre.

There was a significant main effect of time (*p* < 0.001) observed in the physical and physiological variables across all training groups, with ESs ranging from small to large after the completion of the 4-week training period (Figures 2–8).

In addition, the Cr supplementation groups indicated greater adaptations (Group x Time interaction, *p* < 0.001) than the PL group in the CMVJ (Figure 2), 20-m sprint (Figure 3), L-run (Figure 4), peak power (Figure 5) and FI (Figure 7), while no significant differences among the groups were observed in the mean power (*p* = 0.434) (Figure 6) and VO_2max_ (*p* = 0.662) (Figure 8). Interestingly, the CR-Ee and Cr-Hcl groups exhibited greater (%∆) changes compared to the Cr-Mon group in the CMVJ (9.4%, from 34.8 ± 3.1 to 38.1 ± 2.7 cm, [*p* = 0.030] and 9.2%, from 34.7 ± 2.9 to 37.9 ± 2.6 cm, [*p* = 0.043] *versus*, 7.3%, from 34.4 ± 2.5 to 36.9 ± 2.3 cm), 20-m sprint (−5.7%, from 3.88 ± 0.18 to 3.65 ± 0.16 sec, [*p* = 0.045] and −5.6%, from 3.85 ± 0.2 to 3.63 ± 0.16 sec, [*p* = 0.048] *versus*, −4.3%, from 3.87 ± 0.22 to 3.70 ± 0.18 sec), L-run (−6.4%, from 8.6 ± 0.34 to 8.05 ± 0.41 sec, [*p* = 0.015] and −6.2%, from 8.61 ± 0.32 to 8.06 ± 0.41 sec, [*p* = 0.023] *versus*, -4.4%, from 8.61 ± 0.32 to 8.23 ± 0.31 sec), and peak power (10.7%, from 802 ± 59 to 888 ± 72 w [*p* = 0.001] and 10.0%, from 805 ± 60 to 859 ± 58 w [*p* = 0.001] *versus*, 6.8%, from 796 ± 55 to 850 ± 54 w), following the 4-week intervention period, indicating superior outcomes in performance adaptations but not mean power, FI and VO_2max_ after training period.

## 4. Discussion

The research aimed to evaluate the impact of different Cr supplementation formulations in conjunction with a short-term RST intervention during the preparatory phase on the physical and physiological adaptive responses of soccer players. The primary findings reveal that all groups exhibited improvements in physical and physiological performance adaptations, with the supplementation groups demonstrating greater adaptive responses than the PL group in the CMVJ, 20-m sprint, L-run, peak power, and FI. However, no significant differences were noted among the groups regarding the extent of changes in mean power and VO_2max_. Notably, the Cr-Ee and Cr-Hcl formulations showed superior adaptations compared to Cr-Mon in the CMVJ, 20-m sprint, L-run, and peak power, highlighting the advantages of Cr formulations.

Meaningful gains in the CMVJ, 20-m sprint, and L-run performance of soccer players were observed for all training groups following the 4-week intervention, which is in accordance with previous research that reported positive transfer of RST to the bio-motor ability of soccer players [1,3–6] through adaptations in the rate of force development, better muscle fibre synchronisation, and neuromuscular adaptations [30,31]. Furthermore, the groups receiving Cr supplementation demonstrated greater adaptations compared to the PL group, underscoring the advantageous effects of Cr as an ergogenic aid for soccer players in the short-term preparation phase [12,14].

Cr supplementation has been shown to enhance the repeated sprint capabilities of male soccer players [32], potentially by increasing the rate of PCr resynthesis during recovery periods between RST trials [12]. This adaptation might help explain greater involvement of muscle fibres and a delayed onset of fatigue [11,14], leading to more significant improvements in physical performance for the Cr supplementation group compared to the PL group. Interestingly, the Cr-Ee and Cr-Hcl formulations demonstrated greater adaptations compared to Cr-Mon, highlighting the importance of Cr formulation during RST.

Although some manufacturers claim that creatine ethyl ester and creatine hydrochloride may improve solubility and absorption compared with creatine monohydrate, the clinical evidence remains inconsistent [18,19]. In fact, previous research suggests that these newer forms do not necessarily increase intramuscular creatine levels or improve performance more than creatine monohydrate [17]. In addition, there is still no direct evidence in humans showing that creatine hydrochloride has superior bioavailability [18]. Because repeated-sprint training requires maximal effort with short recovery periods [30], the ATP-PCr system is a key performance factor. Given the ongoing debate about the effectiveness of these formulations, further research is needed to determine whether alternative creatine forms offer any ergogenic advantage over creatine monohydrate in improving performance adaptations in soccer players during short-term high-intensity tasks such as the CMVJ, 20-m sprint, and L-run.

RST is widely recognised as an effective training approach for improving anaerobic power output and cardiorespiratory fitness (i.e. VO_2max_) in soccer players, which aligns with our results [1,3–7,25]. The increases in peak and mean power output, as well as VO_2max_, observed after the 4-week RST intervention may stem from adaptations in multiple central and peripheral components triggered by the training [32]. Indeed, changes in oxygen delivery to muscle fibres and enhanced oxygen utilisation by active muscles during aerobic activities, along with improvements in buffering capacity and calcium release, are likely mechanisms underlying the physiological performance adaptations resulting from the RST intervention [3,4,8,28]. More importantly, Cr supplementation has been shown to yield greater adaptations in peak power performance and FI among soccer players than the PL group following a 4-week intervention. The performance of maximal-intensity tasks in the Wingate test is contingent upon the ATP-PCr pathway [30], and the addition of Cr may enhance creatine levels in muscle fibres, leading to improved resynthesis of PCr [12,13] and better muscle fibre recruitment during RST sessions [29], which results in greater adaptations in power performance.

Our findings further suggest that the type of creatine supplement may affect certain adaptations to repeated-sprint training. Although the creatine ethyl ester and creatine hydrochloride groups showed notable improvements in short-term high-intensity tests, these results should be interpreted with caution. While our study supports the potential effectiveness of these formulations, further research is needed to clarify the exact mechanisms, beyond intestinal absorption, that may explain these adaptations. In contrast, no additional improvements were observed among the groups in mean power output or VO_2​max_, suggesting that creatine supplementation may not provide further benefits when performance depends more on other metabolic pathways [32], such as glycolytic and aerobic energy production. Because ATP production during brief sprint efforts of about 5 seconds relies primarily on the ATP-PCr system [8], creatine supplementation may enhance this pathway during repeated-sprint training [28], thereby improving performance in the CMVJ, 20-m sprint, L-run, and peak power output.

However, its effect on longer-duration performance measures, such as mean power output and VO_2​max_, which rely more on glycolytic and aerobic energy systems [33], appears to be limited. These findings suggest that creatine supplementation, particularly in the creatine ethyl ester and creatine hydrochloride forms, may help increase the rate of ATP resynthesis from phosphocreatine during short-term, high-intensity performance tests [16–20]. However, because this study is among the first to compare the effects of creatine monohydrate, creatine ethyl ester, and creatine hydrochloride on the physical and physiological adaptations of soccer players, the findings should be considered preliminary. Further research using direct metabolic markers is needed to clarify the underlying biochemical pathways and intramuscular adaptations associated with these different creatine formulations in athletic populations.

## 5. Study limitations

Several limitations should be considered when interpreting these findings. First, the same 5 g dose was used for all creatine formulations, despite differences in the molar mass of creatine monohydrate, creatine ethyl ester, and creatine hydrochloride. As a result, the exact amount of elemental creatine consumed was not identical across groups. Second, because no loading phase was included, muscle creatine saturation likely developed gradually and may have occurred closer to the end of the 4-week intervention, which could have affected the time available to observe training adaptations. Third, although we proposed that newer formulations such as creatine ethyl ester and creatine hydrochloride might offer pharmacokinetic advantages, such as faster saturation or greater bioavailability, we did not measure intramuscular creatine levels through muscle biopsy or blood analysis. Therefore, any interpretation regarding the mechanistic superiority of a specific formulation remains speculative and should be confirmed in future studies using direct physiological measurements.

## 6. Conclusions

This study aimed to examine the effects of a 4-week RST combined with different formulations of Cr on the physical and physiological performance adaptations in young male soccer players. Our findings indicate that all training groups significantly improved physical and physiological performance adaptations. Furthermore, the addition of Cr supplements during RST led to more substantial enhancements in CMVJ, 20-m sprint, L-run, peak power, and FI compared with the PL condition. Nevertheless, no notable differences were found among the training groups regarding mean power output and VO_2max_. Interestingly, the Ee and Hcl forms of Cr demonstrated more pronounced adaptive responses than the Mon form in CMVJ, 20-m sprint, L-run, and peak power after the 4-week training period.
